# Chemotherapy combined with immune checkpoint inhibitors may overcome the detrimental effect of high neutrophil-to-lymphocyte ratio prior to treatment in esophageal cancer patients

**DOI:** 10.3389/fonc.2024.1449941

**Published:** 2024-10-11

**Authors:** Yuya Hirasawa, Yutaro Kubota, Emiko Mura, Risako Suzuki, Toshiaki Tsurui, Nana Iriguchi, Tomoyuki Ishiguro, Ryotaro Ohkuma, Masahiro Shimokawa, Hirotsugu Ariizumi, Atsushi Horiike, Satoshi Wada, Takeshi Yamashita, Tomotake Ariyoshi, Satoru Goto, Koji Otsuka, Masahiko Murakami, Yuji Kiuchi, Kiyoshi Yoshimura, Takuya Tsunoda

**Affiliations:** ^1^ Division of Medical Oncology, Department of Medicine, Showa University School of Medicine, Tokyo, Japan; ^2^ Division of Medical Pharmacology, Department of Pharmacology, Showa University School of Medicine, Tokyo, Japan; ^3^ Department of Clinical Immuno-Oncology, Clinical Research Institute of Clinical Pharmacology and Therapeutics, Showa University, Tokyo, Japan; ^4^ Department of Clinical Diagnostic Oncology, Clinical Research Institute of Clinical Pharmacology and Therapeutics, Showa University, Tokyo, Japan; ^5^ Showa University Hospital Esophageal Cancer Center, Esophageal Surgery, Tokyo, Japan; ^6^ Pharmacological Research Center, Showa University, Tokyo, Japan

**Keywords:** immune checkpoint inhibitors, immunotherapy, neutrophil-to-lymphocyte ratio, predictive biomarker, esophageal cancer

## Abstract

**Introduction:**

Immune checkpoint inhibitors (ICIs) have emerged as a promising treatment option for esophageal cancer (EC). Although ICIs enable long-term survival in some patients, the efficacy of ICIs varies widely among patients. Therefore, predictive biomarkers are necessary for identifying patients who are most likely to benefit from ICIs to improve the efficacy of the treatment. We retrospectively analyzed the outcomes of combination therapy, including nivolumab plus ipilimumab or chemotherapy plus anti-programmed cell death 1 (PD-1) antibodies in our institute to identify biomarkers.

**Methods:**

Twenty-seven patients received nivolumab plus ipilimumab, and thirty-six patients received chemotherapy plus anti-PD-1 antibodies were included in this study. We analyzed patient characteristics, efficacy, and safety. Multivariable analysis of biomarkers evaluated the correlation among overall survival (OS), progression-free survival (PFS), and the following variables: body mass index, performance status, neutrophil-to-lymphocyte ratio (NLR), C-reactive protein level, and albumin level before treatment.

**Results:**

In multivariable analysis, albumin level was significantly correlated with PFS in the cisplatin plus 5-fluorouracil (CF) plus pembrolizumab group. NLR and albumin level were significantly correlated with OS in the nivolumab plus ipilimumab group. Other variables, including PS, BMI, and CRP did not correlate with any of the outcomes.

**Conclusions:**

High NLR in EC patients prior to treatment was significantly less effective for ICIs. In chemotherapy combined with ICIs, NLR before the treatment was not associated with treatment efficacy, suggesting combination chemotherapy may be beneficial for EC patients with high NLR. NLR may be an indicator of immunocompetence in anti-tumor immunity and a convenient predictive biomarker for selecting appropriate treatments including ICIs.

## Introduction

Esophageal cancer (EC) is one of the most fatal malignancies globally, with limited therapeutic options and a poor prognosis. In last decade, immune checkpoint inhibitors (ICIs) have emerged as a promising treatment option for various kinds of malignancies, including EC. ICIs targeting programmed cell death 1 (PD-1) and cytotoxic T-lymphocyte-associated protein 4 (CTLA-4), have shown promise in improving prognosis in EC patients. Nivolumab plus ipilimumab, or chemotherapy plus anti-PD-1 antibodies, is approved as a first-line treatment for EC (1, 2). However, there are no established criteria to select a first-line treatment. Although ICIs enable long-term survival in some patients, the efficacy of ICIs varies widely among patients. Reliable biomarkers are necessary for identifying patients who are most probable to benefit from ICIs in order to improve the efficacy of the treatment. The role of biomarkers such as PD-L1 expression, microsatellite instability (MSI), and tumor mutation burden (TMB) has been limited in predicting the efficacy of ICIs (3–7). As a convenient predictive biomarker, neutrophil-lymphocyte ratio (NLR) has gained attention as a predictive biomarker for the efficacy of ICIs. Higher NLR prior to the treatment has been consistently associated with poor overall survival (OS) and progression-free survival (PFS) in patients treated with ICIs across various cancer types, including EC (8–18). The underlying mechanisms involve the role of neutrophils in the tumor microenvironment (TME). Neutrophils can promote tumor progression and suppress T cell-mediated anti-tumor responses, which may explain the association between higher NLR and poor therapeutic outcomes (19–21). These findings suggest that NLR, a cost-effective and accessible biomarker, may play an important role in selecting appropriate first-line treatment, including ICIs. We retrospectively analyzed the patients’ baseline characteristics, efficacy, and safety of first-line treatment for EC in clinical practice and analyzed the predictive biomarker to identify the patients who benefit from ICIs. In addition, the immune system is impaired with age due to the phenomenon of immunosenescence, which includes a decrease in naïve T cells, an increase in memory T cells, reduced immune responsiveness, and inflammation caused by senescent T cells. To evaluate whether NLR and treatment efficacy are affected by immunosenescence, we conducted a stratified analysis of NLR and the best overall response (BOR) between patients under 65 years and those over 65 years in each treatment group (22–27).

## Materials and methods

Patients who received ICIs for pathologically confirmed EC between February 2020 and June 2023 were included in this study. Twenty-seven patients were administered nivolumab plus ipilimumab, and thirty-six patients were administered cisplatin plus 5-fluorouracil (CF) plus pembrolizumab. We conducted a retrospective analysis to evaluate the clinical effectiveness, safety, and clinical laboratory data of the patients. The data collection focused on routine medical care, involving patient characteristics such as age, sex, ECOG performance status (PS), body mass index (BMI), stage, and histology. Laboratory findings, imaging studies, and assessments of safety and efficacy were also included. Biomarker analysis involved ECOG PS, BMI, neutrophil-to-lymphocyte ratio (NLR), C-reactive protein (CRP), and albumin level prior to the treatment. Safety evaluation was based on the presence and severity of immune-related adverse events (irAEs) according to the Common Terminology Criteria for Adverse Events (CTCAE) version 5.0. Efficacy was measured according to the Response Evaluation Criteria in Solid Tumors (RECIST) ver 1.1, which measured the overall response rate (ORR), disease control rate (DCR), progression-free survival (PFS), and overall survival (OS).

The studies involving human participants were reviewed and approved by the Ethics Committee of Showa University School of Medicine (approval No. 22-168-A). Informed consent was obtained through an opt-out method on the website since this is a retrospective study using only medical records. We ensured a proper opportunity to refuse the use of medical records.

### Statistical analysis for biomarkers

Efficacy was assessed according to OS and PFS. Variables included in the multivariable analysis were PS (0-1 vs. 2-3), BMI, NLR, CRP, and albumin level, all measured before treatment. Multivariable analyses were conducted using the Cox proportional hazards model for both OS and PFS. Survival time analysis was performed by plotting survival curves using the Kaplan–Meier method, which were compared using the log-rank test. Two-sided p-values less than 0.05 were considered statistically significant. Statistical analyses were conducted using JMP ^®^ Pro 15(SAS Institute Inc., Cary, NC, USA).

## Results

### Baseline characteristics of the patients

The demographic and clinical features of the patients are showed in **Table 1**. The median patient age was 69 years (range 46–85 years), 80.6% were men, and 90.5% of the cases were ECOG PS 0-1, whereas 7.9% and 1.6% of the patients had PS 2 and 3, respectively. Stage IVB occurred in 95.2% of the cases. All patients were diagnosed with squamous cell carcinoma of the esophagus. As for the treatment, 58.7% were first-line treatment, while 31.7% were second-line treatment. The reasons for administering the treatment as a second-line treatment were approximately equally divided: half of the cases involved patients who were deemed unresectable after neoadjuvant chemotherapy, and the other half involved patients who initiated treatment following chemoradiotherapy. PD-L1 expression was examined in approximately 54.0% of patients. There were no notable findings in pretreatment BMI, neutrophil count, lymphocyte count, NLR, CRP, or albumin levels. NLR was not higher in patients over 65 years than in those under 65 years. Conversely, in the nivolumab plus ipilimumab group, NLR was higher in patients under 65 years than in those over 65 years.

**Table 1:** Baseline characteristic of the patients (n=63).

Characteristic	Nivolumab+Ipilimumab (n=27)		CF+Pembrolizumab (n=36)
Median Age – yr (range)	74 (49-85)		67 (46-80)
Sex – no (%)			
Male	20 (74.1)		31 (86.1)
Female	7 (25.9)		5 (13.9)
BMI – mean (95% CI)	18.8 (15.6-29.8)		21.4 (15.6-29.7)
ECOG PS – no (%)			
0	1 (3.7)		3 (8.4)
1	22 (81.5)		31 (86.1)
2	3 (11.1)		2 (5.6)
3	1 (3.7)		0 (0.0)
Stage – no (%)			
III	1 (3.7)		0 (0)
IVA	1 (3.7)		1 (2.7)
IVB	25 (92.6)		35 (97.3)
Pathological Diagnosis – no (%)			
Squamous Cell Carcinoma	27 (100)		36 (100)
Lines of treatment – no (%)			
1st line	12 (44.5)		25 (69.4)
2nd line	10 (37.0)		10 (27.8)
3rd line	3 (11.1)		1 (2.8)
4th line	2 (7.4)		0 (0.0)
PD-L1 expression – no (%)	[28-8]		[22C3]
<1% or <10	0 (0.0)		4 (11.1)
>1% or >10	11 (40.7)		19 (52.8)
NA	16 (59.3)		13 (36.1)
Clinical variables (prior to treatment) – median (IQR)			
Neutrophil Count (/μL)	3590 (1930-9860)		3630 (1540-8970)
Lymphocyte Count (/μL)	800 (300-1700)		1080 (240-4820)
NLR (ratio)	3.9 (1.9-19.0)		2.9 (1.2-14.5)
CRP (mg/dL)	0.38 (<0.04-7.85)		0.31 (<0.04-4.68)
Albumin level (g/dL)	3.8 (2.5-4.6)		3.8 (2.6-4.5)
NLR (ratio) - median (IQR)			
Age < 65yr		4.88 (3.40-8.30)	3.30 (2.40-4.40)
Age ≥ 65yr		3.51 (2.54-10.52)	3.30 (2.10-6.40)

ECOG, Eastern Cooperative Oncology Group; PS, Performance status; CRP, C-reactive protein; NLR, Neutrophil-to-lymphocyte ratio; IQR, interquartile range; CF, cisplatin plus 5-fluorouracil.

### Response to treatment and safety with nivolumab plus ipilimumab

**Figure 1:**
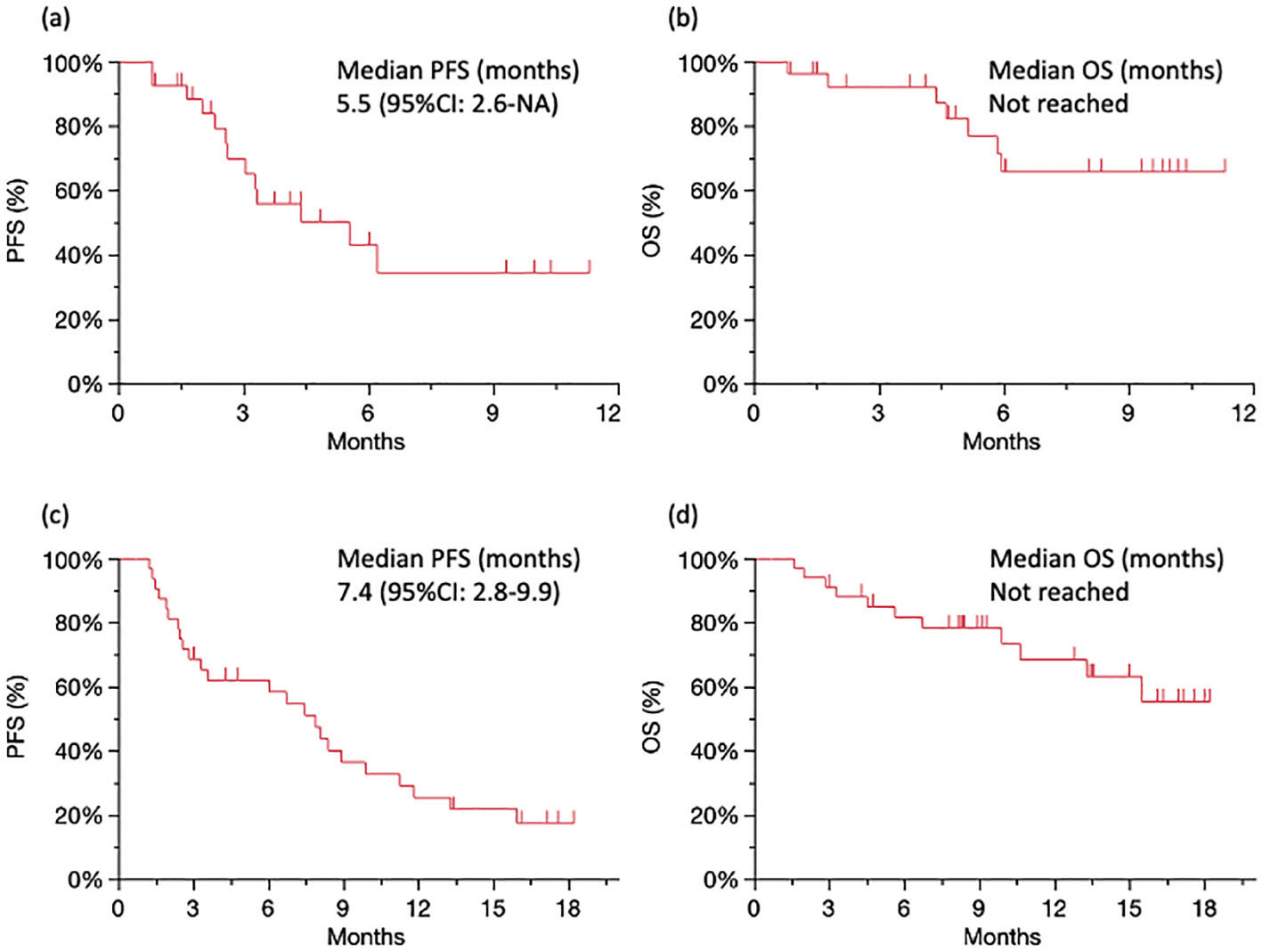
Survival analysis Kaplan–Meier curves of PFS **(A)** and OS **(B)** for patients treated with nivolumab plus ipilimumab. Kaplan–Meier curves of PFS **(C)** and OS **(D)** for patients treated with CF plus pembrolizumab. PFS, progression-free survival; OS, overall survival.

The median PFS was 5.5 months (95% confidence interval [CI]: 2.6–NA; **Figure 1A**), and the median OS was not reached (**Figure 1B**) for patients treated with nivolumab plus ipilimumab. The best overall response (BOR) was 18.5% for partial response (PR), 33.3% for stable disease (SD), and 37.0% for progressive disease (PD), respectively. The ORR was 18.5% and the DCR was 55.5% (**Table 2A**). There were five cases of PR in patients over 65 years. IrAEs were observed in 51.8% of cases of all grades and 14.8% in grades 3–4 with nivolumab plus ipilimumab (**Table 2B**).

**Table 2A:** Response to treatment.

Result	Nivolumab+Ipilimumab (n=27)	CF+Pembrolizumab (n=36)
Median follow-up time – months (95% CI)	5.8 (0.8-11.3)	9.0 (0.7-18.2)
Best Overall Response – no (%)		
CR	0 (0)	0 (0)
PR	5 (18.5)	11 (30.6)
SD	9 (33.3)	12 (33.3)
PD	10 (37.0)	10 (27.8)
NA	3 (11.1)	3 (8.3)
Age < 65yr		
CR	0 (0)	0 (0)
PR	0 (0)	5 (13.9)
SD	3 (11.1)	6 (16.7)
PD	1 (3.7)	0 (0)
NA	1 (3.7)	2 (5.5)
Age ≥ 65yr		
CR	0 (0)	0 (0)
PR	5 (18.5)	6 (16.7)
SD	6 (22.2)	6 (16.7)
PD	9 (33.3)	10 (27.8)
NA	2 (7.5)	1 (2.7)
ORR (%)	18.5	30.6
DCR (%)	55.5	63.9

CR, Complete Response; PR, Partial Response; SD, Stable Disease;

PD, Progressive Disease; ORR, Overall Response Rate; DCR, Disease Control Rate.

**Table 2B:** Immune-related adverse events.

irAEs	Nivolumab+Ipilimumab (n=27)	CF+Pembrolizumab (n=36)
All Grade	14 (51.8)	11 (30.5)
Grade 3-4	4 (14.8)	4 (11.1)

### Response to treatment and safety with chemotherapy plus pembrolizumab

The median PFS was 7.4 months (95% CI: 2.8–9.9; **Figure 1C**), and the median OS was not reached (**Figure 1D**) for patients treated with CF plus pembrolizumab. The BOR was 30.6% for PR, 33.3% for SD, and 27.8% for PD, respectively. The ORR was 30.6% and the DCR was 63.9% (**Table 2A**). There were no cases of PD in 13 patients under 65 years. IrAEs were observed in 30.5% of cases of all grades and 11.1% in grades 3–4 with CF plus pembrolizumab (**Table 2B**).

### Multivariable analysis of biomarkers

Multivariable analysis was performed to determine predictive biomarkers from routine clinical data. Albumin level, not NLR, was significantly correlated with PFS in the CF plus pembrolizumab group (**Table 3A**). NLR and albumin level were significantly correlated with OS in the nivolumab plus ipilimumab group (**Table 3B**). Other variables, including PS, BMI, and CRP did not correlate with any of the outcomes.

**Table 3A:** Cox proportional hazards analysis for PFS.

PFS	Multivariable analysis					
	Nivolumab+Ipilimumab (n=27)			CF+Pembrolizumab (n=36)		
Variables	Hazard ratio	95% CI	p-value	Hazard ratio	95% CI	p-value
PS (0-1 vs 2-3)	1.05	0.14-8.18	0.96	0.26	0.03-2.27	0.23
BMI (/unit)	1.10	0.93-1.30	0.24	1.01	0.90-1.14	0.81
CRP (/unit)	1.04	0.58-2.00	0.91	0.72	0.31-1.67	0.44
NLR (/unit)	1.02	0.81-1.19	0.81	0.99	0.78-1.27	0.97
Albumin level (/unit)	0.26	0.09-2.27	0.28	0.16	0.03-0.80	0.025

PFS was significantly correlated with albumin level in the CF plus Pembrolizumab group by multivariable analysis.

**Table 3B:** Cox proportional hazards analysis for OS.

OS	Multivariable analysis					
	Nivolumab+Ipilimumab (n=27)			CF+Pembrolizumab (n=36)		
Variables	Hazard ratio	95% CI	p-value	Hazard ratio	95% CI	p-value
PS (0-1 vs 2-3)	0.29	0.01-4.60	0.38	1.37	0.01-193.36	0.90
BMI (/unit)	0.99	0.62-1.67	0.98	1.03	0.78-1.28	0.80
CRP (/unit)	2.89	0.73-15.91	0.15	1.94	0.48-12.00	0.41
NLR (/unit)	1.22	1.02-1.16	0.049	0.89	0.58-1.25	0.54
Albumin level (/unit)	0.005	0.0006-0.41	0.049	0.18	0.02-1.47	0.13

OS was significantly correlated with NLR and Albumin level in the Nivolumab plus Ipilimumab group, not CF plus Pembrolizumab group, by multivariable analysis.

## Discussion

Predictive biomarker analysis was demonstrated that NLR before the treatment was significantly correlated with OS in the nivolumab plus ipilimumab group. Conversely, NLR was not significantly correlated with efficacy in the CF plus pembrolizumab group. We focused on NLR because it has been reported to be associated with immunological response.

As for biomarkers, the function of PD-L1 expression, MSI, and TMB as biomarkers in predicting the efficacy of ICIs is uncertain, because ICIs are ineffective for some patients with these biomarkers (3–7). Gut microbiota, cytokine signatures, and tertiary lymphoid structure (TLS) have all been proposed as biomarkers, but they have not yet been authorized in clinical practice (28–34).

In aspects of NLR as a predictive biomarker, previous reports have shown that a high NLR prior to treatment correlates with poor clinical efficacy in patients with various solid tumors, such as pancreatic, lung, and colorectal tumors, treated with chemotherapy (35–37). Patients with higher pretreatment NLR also demonstrated poor clinical response to ICIs in various cancer types, including EC, gastric cancer (GC), non-small cell lung cancer (NSCLC), malignant melanoma, urothelial carcinoma, renal cell carcinoma, respectively (8–18). A retrospective cohort study of 1714 patients with 16 different cancer types also showed that higher NLR is significantly associated with poor overall survival (OS) and progression-free survival (PFS) with ICIs treatment (38). However, some reports have demonstrated no correlation between NLR and clinical efficacy; thus, these results are still controversial (39). Caution is required in its evaluation and utilization of NLR, since the cutoff values vary, and its availability as a biomarker varies by cancer type and treatment (40, 41). The underlying mechanisms responsible for the association between higher NLR and poor therapeutic outcomes involves the role of neutrophils in the tumor microenvironment. Neutrophils can promote tumor progression and suppress T cell-mediated anti-tumor responses (19–21).

NLR is typically elevated, reflecting chronic stress and inflammation. NLR tends to increase with aging in many vertebrates, including humans (42). Conversely, the bigmouth buffalo (*Ictiobus cyprinellus*), a long-living fish, has been reported to show a negative correlation between age and NLR (43). This finding suggests that a low NLR may be indicative of good health conditions, characterized by low levels of stress and inflammation. In aspects of cancer, neutrophils are elevated by immunosuppressive mediators, primarily IL-6 derived from tumors. Tumor-derived IL-6 has been shown to be significantly associated with tumor size, stage, and proliferative activity as measured by Ki-67; as a result, it will probably increase with the disease progresses (44, 45). IL-6 stimulates the bone marrow and increase the number of neutrophils. Additionally, tumor-derived IL-8 stimulates neutrophil migration to the tumor sites (46). Neutrophils secrete matrix metalloproteinase-8 (MMP-8), vascular endothelial growth factor (VEGF), and neutrophil elastase, which enhance tumor growth, angiogenesis, and suppression of T cells activity in the TME (19–21). Thus, neutrophils are considered to promote tumor progression and are referred to as tumor-associated neutrophils (TANs) (47). It is remarkable that TANs suppress activity of T cells in TME (48, 49). In contrast, the lymphocyte count is a key factor in antitumor immunity. Thus, low NLR which implies a lymphocyte-dominant condition may be an indicator of high immunocompetence in anti-tumor immunity. Furthermore, a correlation has been reported between the therapeutic efficacy of ICIs and the presence of TLS in tumor tissue, and a positive correlation has been demonstrated between low NLR and high TLS expression (50, 51). On the other hand, favorable ICI responses have been demonstrated with high intra-tumoral CD8-to-neutrophil ratios, while low ratios predicted ICI treatment failures and poor progression-free survival (52). These findings suggest that a neutrophil-dominant conditions, including high NLR, are in favor of tumors.

Conversely, NLR was not significantly correlated with efficacy in the CF plus pembrolizumab group, although ICIs were included in the treatment. The underlying mechanisms may involve chemotherapy-induced bone marrow suppression. Chemotherapy-induced bone marrow suppression mainly reduces neutrophil counts. These findings suggest that chemotherapy may reduce the detrimental effects of neutrophils on anti-tumor immunity, such as some suppression of T-cell mediated anti-tumor responses in the TME. It is suggested that the combination of chemotherapy may create TME favorable to the effect of ICIs and might be beneficial for EC patients with high NLR.

A limitation of this study is that it was a retrospective analysis conducted at a single institute. Another limitation is that the observation period was not long enough, and the number of patients involved in this study was relatively small. It is necessary to confirm these findings in a prospective study.

The main purpose of this manuscript was to describe NLR as a predictive biomarker. Further analysis will be performed to evaluate the difference between pre-treatment and post-treatment NLR values in patients treated with nivolumab plus Ipilimumab vs patients treated with chemotherapy plus pembrolizumab and to evaluate if these differences are statistically significant and if they correlate with the disease response. In addition, data regarding the utility of post-treatment NLR as a biomarker, including not only BOR but also analysis of OS and PFS will be presented in a new manuscript.

## Conclusion

High NLR in patients with EC prior to the treatment may be less effective for ICIs. In chemotherapy combined with ICIs, NLR at pre-treatment was not associated with clinical efficacy, suggesting that combination chemotherapy may be beneficial for EC patients with high NLR before the treatment. NLR might be an indicator of immunocompetence in antitumor immunity and a convenient predictive biomarker for selecting appropriate treatment including ICIs in daily practice.

## Data Availability

The raw data supporting the conclusions of this article will be made available by the authors, without undue reservation.
